# Risk factors of cholangiocarcinoma: more than control of liver fluke

**DOI:** 10.2478/abm-2024-0026

**Published:** 2024-10-31

**Authors:** Editorial Office of Asian Biomedicine

**Affiliations:** Faculty of Medicine, Chulalongkorn University, Bangkok 10330, Thailand

Cholangiocarcinoma (CCA) encompasses cancers found in different parts of biliary tree, comprising intrahepatic, perihilar, or extrahepatic (distal) parts. CCA does not include cancer of gallbladder or ampulla of vater. It is a heterogeneous disease probably resulting from a complex interaction between genetic, environmental, and behavioral risks of individuals living in different parts of the world [1]. The typical occurrence of CCA is between 50 years and 70 years. CCA is an aggressive cancer and most patients do not survive beyond 5 years after diagnosis. Liver transplantation is a treatment option for those who satisfy appropriate inclusion and exclusion criteria [2].

In many CCA patients, the specific risk factors unique to individuals cannot be ascertained [1]. Therefore, there may be other underlying risks of CCA yet to be defined [1]. Indeed, the staging and prognosis of CCA are elusive and depend on the anatomical location of the tumors (intrahepatic, perihilar, and distal bile duct tumor) [2].

A number of risk factors for CCA have been recognized. Asia has a very high incidence of CCA, possibly due to a high prevalence of hepatobiliary flukes [3, 4]. A species of liver fluke, *Clonorchis sinensis*, has been prevalent in China and Northern Vietnam while *Opisthorchis viverrini* has prevailed in many Southeast Asian nations [5]. Thus, the epidemiological information highlights the role of specific climatic, environmental, and lifestyle factors in influencing the geographical distribution of *C. sinensis* and *O. viverrini*. In the United States, CCA has been identified in less than 10% of all liver cancers [6]. Nevertheless, a specific risk factor cannot be established in many patients.

The main risk factors for CCA in the United States and Europe are primary sclerosing cholangitis and choledochal cysts (fibropolycystic liver disease) [6]. Many large population-based studies have identified a number of risk factors. It is recognized that inflammation and cholestasis might be the driving forces in CCA development. Therefore, the current view is that cholestatic liver diseases (e.g., primary sclerosing cholangitis and fibropolycystic liver diseases), liver cirrhosis, and biliary stone disease increase the risk of CCA. Certain infections such as hepatitis B and C and liver flukes can also enhance CCA risk [7]. Other risk factors include inflammation (chronic pancreatitis), intrahepatic stone disease, alcohol, tobacco, toxic substances such as nitrosamine, chronic diseases (diabetes, obesity, and non-alcoholic fatty liver disease), cirrhosis, and a number of genetic disorders [7]. In terms of histopathology, the majority of CCA are adenocarcinoma [8].

Teerasarntipan et al. in this issue reported the prevalence of CCA in areas not endemic for liver fluke infection [9]. They concluded that *O. viverrini* infection has become trivial contribution to CCA development in non-endemic areas of liver fluke. Conversely, smoking and chronic biliary tract disorders are major risk factors for CCA [9]. Therefore, identification of the strategies to curb modifiable risk factors such as lifestyle and environmental parameters are actually not overemphasized and should also capture our attention to prevent CCA, particularly in non-endemic areas of liver fluke. It should also be realized that while the factors outlined above can increase the risk of CCA, the exact determinants of CCA often remains to be established in many patients. Different patients with CCA may have different degrees of contributions from genetic, lifestyle, and environmental risk factors.
